# COACH2MOVE: THE STORY OF IMPLEMENTING A PERSON-CENTERED PHYSICAL THERAPY APPROACH IN CLINICAL PRACTICE

**DOI:** 10.1093/geroni/igac059.066

**Published:** 2022-12-20

**Authors:** Ward Heij, Lieke Sweerts, Bart Staal, Philip van der Wees, Ria Nijhuis- van der Sanden, Thomas Hoogeboom

**Affiliations:** University of Utah, Salt Lake City, Utah, United States; Radboud university medical center, Nijmegen, Gelderland, Netherlands; Radboud university medical center, Nijmegen, Gelderland, Netherlands; Radboud university medical center, Nijmegen, Gelderland, Netherlands; Radboud university medical center, Nijmegen, Gelderland, Netherlands; Radboud university medical center, Nijmegen, Gelderland, Netherlands

## Abstract

Coach2Move is a person-centered physical therapy approach to promote physical activity among community-dwelling older adults, which consists of motivational interviewing and other motivational strategies. Coach2Move has been implemented in the Netherlands with 52 physical therapists and 294 patients in 16 practices. Implementation has led to better outcomes (increased physical activity, improved functional mobility, and lower levels of frailty) in fewer treatment sessions compared with usual care physical therapy treatment. Implementing was carried out using the following steps: 1) an e-assessment to determine the baseline level of knowledge regarding Coach2Move of the participating therapists; 2) two education days in Motivational Interviewing and Coach2Move specific motivational strategies; 3) three unique peer-assessment meetings and 4) a retest of the e-assessment one year later. We will discuss our rationale for selecting implementation strategies and which barriers and facilitators we have experienced in implementing successful scientific research in daily clinical practice.

